# Mutation analysis of annual sediment discharge at Wu Long station in Wu Jiang River Basin from 1960 to 2016

**DOI:** 10.1371/journal.pone.0225935

**Published:** 2019-12-16

**Authors:** Peng Chen, Guangming Tan, Jinyun Deng, Quanxi Xu, Rouxin Tang

**Affiliations:** 1 State Key Laboratory of Water Resources and Hydropower Engineering Science, Wuhan University, Wuhan, China; 2 Key Lab. of River Regulation and Flood Control of Ministry of Water Resources, Changjiang River Scientific Research Inst., Wuhan, China; 3 Bureau of Hydrology, Yangtze River Water Resources Commission, Wuhan, China; Ghent University, BELGIUM

## Abstract

This article introduces a new method for mutation detection, the approximate entropy method, which is based on the complexity of time series. The mutation of average annual sediment discharge in Wu Jiang River Basin from 1960 to 2016 is detected by the introduced approximate entropy method, and compared with the results of double cumulative curve method and B-G segmentation algorithm. The mutation physical mechanism of the sediment discharge is discussed from the aspects of sediment source, annual distribution and interannual variation, climate change, impact of water conservancy and water conservation projects on sediment transport. The results show that mutation points occurred at 1984 and 2008 at Wu Long station, and the sediment discharge has a significant change after 2008. The mutation of average annual sediment discharge in Wu Jiang River Basin is caused by both climate change and human activity. Sediment reduction effect of the hydraulic engineerings built since 1990s climate is main and direct, and the climate change have secondary effect on sediment discharge change.

## Introduction

Knowledge of the transfer of sediment through river systems is essential for understanding the physical, chemical and biological processes on the Earth’s surface [1]. The factors affecting sediment yield and sediment transport can be divided into natural and anthropogenic [2,3,4]. Among these factors, human activities have significantly altered global river sediment transport regimes across the planet [5,6].

The Yangtze River is the longest river in China with a catchment area of 1.8×10^6^ km^2^. The river can be divided into two parts: the upper stream above the city of Yichang, and the mid-lower stream from Yichang to the ocean. The annual water discharge and suspended sediment discharge (SSD) at the beginning of the estuary was high between 1950 and 2000, with an average value of 905.1×10^12^ m^3^/y and 433×10^6^ t/y, respectively [7]. However, since the TGD (the largest dam in the world) was constructed in the upper stream in 2003, the SSD into the Yangtze estuary has decreased nearly 70% [8,9]. A number of studies focused on the yearly changes in SSD from 1951 to 2000s [10,11], on the fate of suspended sediments delivered to the East China Sea [12], on the many impacts of such a decline in SSD, on the relation between SSD and water discharge [13], and on the effects of extreme droughts on SSD [14]. The suspended sediment concentration (SSC) in the Yangtze River is dominated by discharge, deforestation, land use, soil erosion, and dam impoundment. Over the period ranging from 1956 to 2013, soil erosion induces high SSC, which gradually decreases because of the implementation of soil and water conservation strategies [15]. Therefore, it is urgent to find out the mutation year of sediment discharge, clarify the influencing factors of river sediment transport system and quantify the extent of its influence on sediment transport process in Yangtze River Basin.

The traditional methods for studying water and sediment mutation include double cumulative curve method, ordered cluster analysis method and Mann-Kendall rank test method [16,17]. These methods have their own characteristics and can show the mutation feature of water and sediment to varying degrees. However, owing to the detection results of the above methods are determined by the choice of time scale, and the detected mutation points have multi time scale characteristics [18], so there are some defects in the processing of nonlinear and non-stationary time series data [19,20].

In the 1990s, Pincus proposed and developed the concept of approximate entropy (denoted as ApEn) from the perspective of measuring the complexity of time series [21,22,23]. Approximate Entropy is an ideal non-linear dynamic detection method. It is widely used in the diagnosis of medical and mechanical equipment faults because of its advantages of short data amount, better anti-noise and anti-jamming ability, and can be used for both random and deterministic signals [24,25]. The discipline of river dynamics is a complex, non-linear, time-space dynamic system. Sediment movement and river bed evolution have continuity and correlation in space-time evolution. On the basis of introducing ApEn and its physical significance, this paper applies ApEn to the mutation detection of ideal time series in annual sediment transport, analyses the changes of main factors affecting sediment transport before and after mutation, and explores the physical background of mutation in sediment transport, which is of great significance for predicting the law of sediment transport in rivers and its evolution trend in the future.

## Materials and methods

### Materials

**Fig 1:**
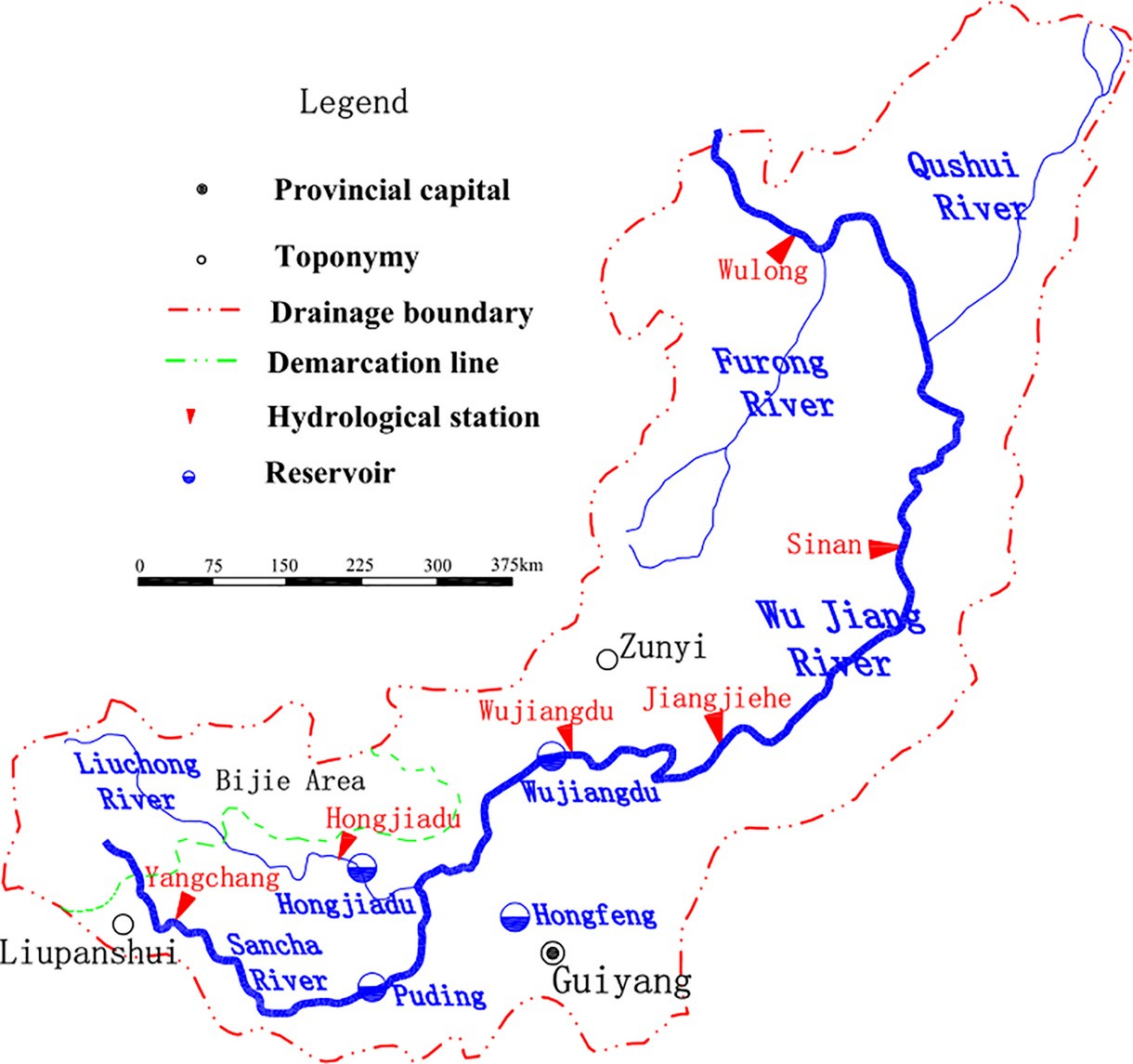
Distribution chart of main hydrometric stations and large hydropower stations in Wu Jiang Basin.

Wu Jiang River is one of the important tributaries in the upper Yangtze River. Its catchment area is 87,920 km^2^, the river length is over 1,030 km, and the natural drop is over 2,120 m. The Wu Jiang River system is feathered with 58 first-class tributaries. The Wu Jiang River Basin is mostly located in the slope zone of the transition from the northeastern Yunnan-Guizhou Plateau to the western Hunan hills, mainly in mountainous areas. Rainfall in the basin is mainly concentrated in May to September, with more autumn rainfall and uneven distribution of rainfall in the basin. The Wu Jiang River Valley is severely cut down and has a "V" shape. Only in some parts of the valley are wide valleys composed of sand and shale. The Wu Long hydrological station is seated in Wu Long County of Chongqing City, located at 107 45 E and 29 19 N, and the lower reaches of Fuling to the Yangtze River about 60 kilometers downstream. It is the control station of Wu Jiang to the Yangtze River (Fig 1).

The data on the annual sediment discharge for the 1960–2016 period at the Wu Long hydrological station was obtained from the Changjiang Water Resources Committee (CWRC) and the Changjiang Sediment Bulletins. The changing trends and mutation points of the sediment discharge series at the Wu Long hydrological station was detected according to the ApEn method.

## Method

ApEn is a method of distribution statistics to quantify the complexity of time sequence based on marginal probability. The brief introduction of its specific algorithm below:

Suppose there are time series u (1), u (2), ⋯, u (N) at a length of N. Construct a group of vector quantity X (1), X (2), ⋯, X (N-m+1) with dimensionality as m, among which,

X(i)={u(i),u(i+1),⋯,u(i+m−1)},i=1,2,⋯,N−m+1 (1)

Define d [X (i), X (j)], the distance between X (i) and X (j) as the one with the largest difference value in corresponding elements, that is,

d[X(i),X(j)]=max[|u(i+k)−u(j+k)|],k=0,1,⋯,m−1 (2)

For each i (1≤i≤N-m+1), define

$$C_{i}^{m}(r)=\{d[X(i),\;X(j)]\leq number\;of\;r\}/(N-m+1)$$ (3)

$C_{i}^{m}(r)$ refers to the probability that d [X (i), X (j)], the distance between vector X (j) and X (i), is less than r when dimensionality is m and allowable deviation is r. Thus, it can show the degree of approximation between X (j) and X (i), that is correlation degree.

Take the logarithm of $C_{i}^{m}(r)$, and then get the average value of all i. Take it as φ^m^(r). Then

$$\emptyset^{m}(r)=\frac{1}{N-M+1}\sum_{i=1}^{N-m+1}l_{n}C_{i}^{m}(r)$$ (4)

Add 1 to m, and repeat the above procedure, then get $C_{i}^{m+1}(r)$ and φ^m+1^(r). Theoretically, the approximate entropy of this series is

$$ApEn(m,r)=\lim_{N\to \infty }\left[\emptyset^{m}(r)-\emptyset^{m+1}(r)\right]$$ (5)

Generally speaking, this limit exists with probability 1. In practical work, N is impossible to be ∞. When N is a limited value, we can get the estimated value of ApEn as below:

$$ApEn(m,r)=\emptyset^{m}(r)-\emptyset^{m+1}(r)$$ (6)

Apparently, the value of ApEn is related to the value of m and r. According to actual practice, Pincus suggests taking m = 2 and r = 0.1–0.2σ (σ is the standard deviation of original data). In this article, m = 2 and r = 0.15σ.

From the above deduction process, it is clear that the ApEn method reflects the approximation degree between the two modes of the sequence in the m-dimensional case, and the possibility of generating new modes when the dimension changes. The larger the ApEn, the greater the probability of generating a new pattern, the more complex the sequence, and the worse the predictability of the system. The ApEn method gives the case that the incidence of new patterns increases or decreases with the dimension, thus reflecting the structural complexity of the sequence. At the same time, it does not attempt to completely reconstruct the attractor, so it has good applicability to various non-linear time series.

## Results

### Sediment discharge mutation detection

**Fig 2:**
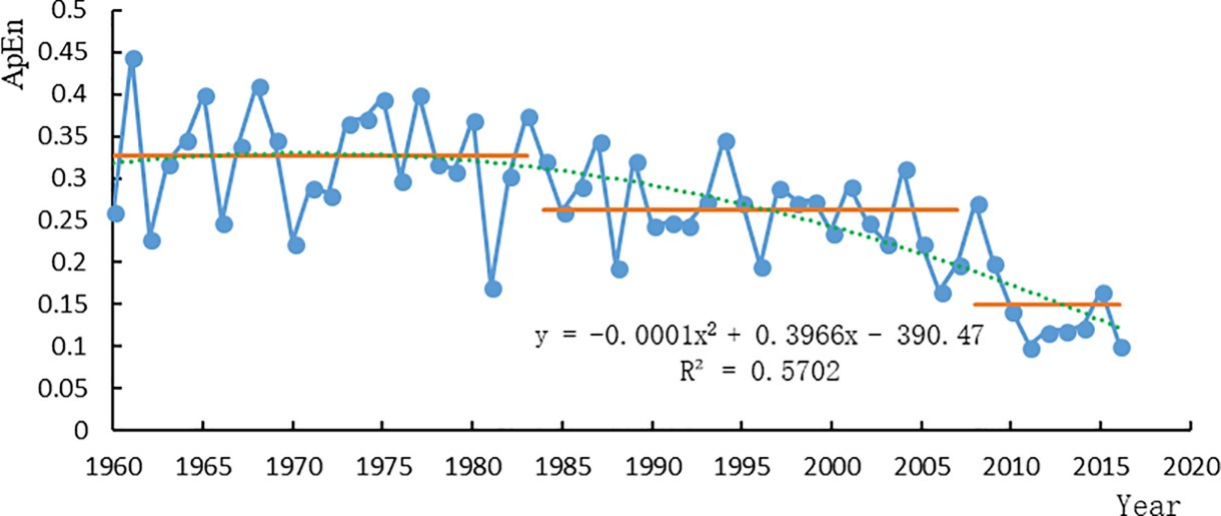
ApEn value process line of annual sediment discharge at Wu Long Station.

**Fig 3:**
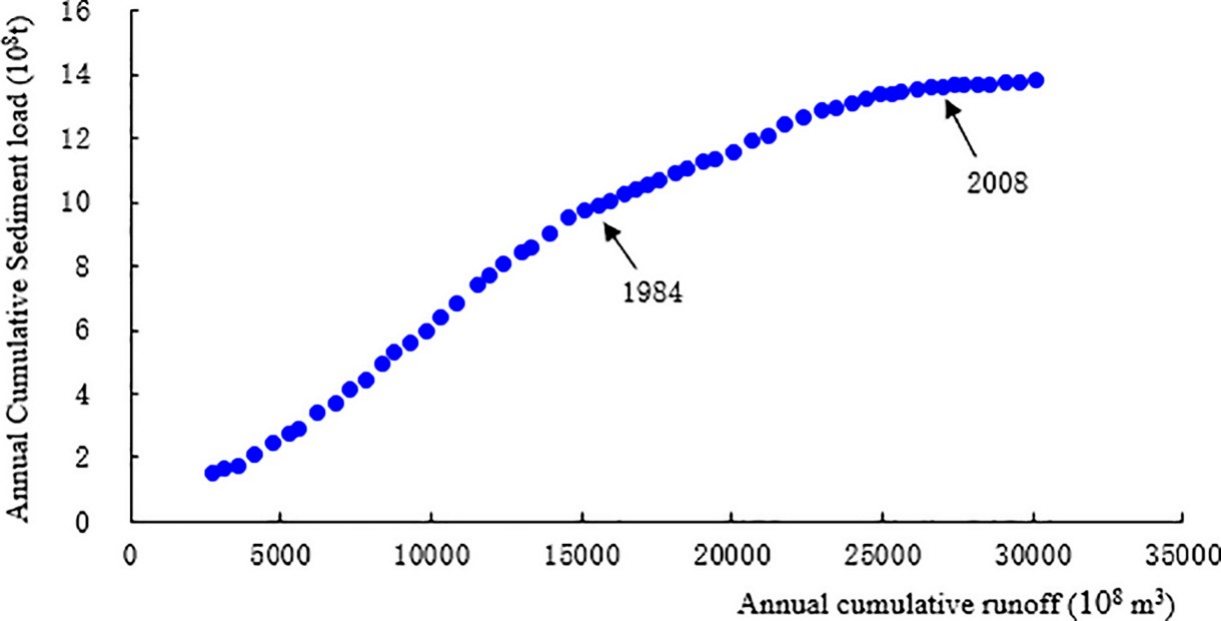
Double cumulative graph of annual runoff and sediment discharge at Wu Long Station.

The data are from Wu Long station in Wu Jiang River Basin for 57 years from 1960 to 2016. Based on the ApEn method, the mutation year of sediment discharge time series during 57 years at Wu Long station is detected, and the significance level of ApEn annual sediment discharge is tested by sliding t test (Confidence level was |T_0.05/2_| = 2) (Table 1). The detection results show that the annual sediment discharge has undergone two mutations in recent 57 years at Wu Long station of Wu Jiang River, occurred in 1984 and 2008, and the ApEn significant levels of sediment discharge in 1984 and 2008 are -2.113 and -4.308, respectively, which have passed the test. Sediment transport time series can be divided into three segments in Wu Jiang River according to mutation points, that is, from 1960 to 1983, 1984 to 2007 and 2008 to 2016 (Fig 2). Meanwhile, if the water and sediment characteristics of the basin change systematically, there will be an obvious turning point on the double cumulative curve of water and sediment discharge, that is, the slope of the cumulative curve will obviously increase or decrease. According to the double cumulative curve of water and sediment at Wu Long station, it can be seen that Wu Long station in the Wu Liang River experienced a turning point in 1984 and 2008, and the sediment discharge decreased significantly (Fig 3).

**Table 1:** Detection results of ApEn on annual sediment discharge at Wu Long station by sliding t.

Mutation point detection	Year 1984	Year 2004	Year 2008
|T_0.05/2_|	2	2	2
T_m_	-2.113	-1.879	-4.308
pass the test or not	Y	N	Y

B-G segmentation algorithm method was firstly put forward to detect mutations in non-linear and non-stationary time series by Bernaola-Galavan in 2001 [26]. According to B-G segmentation algorithm, the mutation of 57-year low-pass filtering time series of sediment discharge at Wu Long station in Wu Jiang River are detected. The test results show that, the annual sediment discharge has undergone two major mutations in the past 60 years, 1984 and 2008 respectively (Table 2). First, mutation was detected in 1984 (significant level 0.997); mutation detection was carried out in 1960–1983 and 1984–2016 for sub-sequences. By significance level test, the mutation point was found in 2008, and the significance level was 0.963.

**Table 2:** Detection results of mutation year by B-G segmentation algorithm.

Mutation Point Detection	Year 1984	Year 2004	Year 2008
T_m_	2.93	1.31	2.56
P(T_m_)	0.997	0.576	0.963
pass the test or not	Y	N	Y

**Fig 4:**
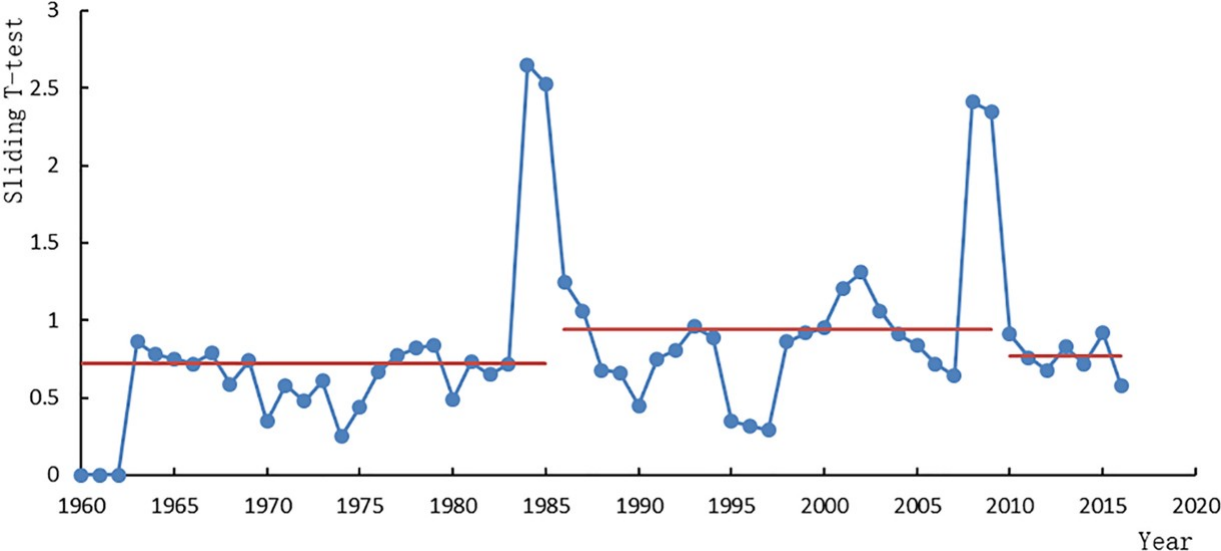
Sliding T-test of annual sediment discharge at Wu Long Station.

By comparing the ApEn method with the B-G segmentation algorithm method and sliding t test method (Fig 4), it can be seen that although the B-G segmentation algorithm method and sliding t test method can also detect the corresponding structural mutations, it cannot reflect the dynamic structural differences and complexity before and after the mutations. The ApEn method can reflect the degree of self-similarity of time series in patterns and the possibility of generating new patterns when the dimension m changes. The larger the ApEn value, the greater the probability of generating a new pattern, the more complex the sequence, and the worse the predictability of the system. It gives the case that the incidence of new patterns increases or decreases with the dimension, thus reflecting the structural complexity of the data. Therefore, the ApEn method has obvious advantages in mutation detection of annual sediment discharge in river basins, and it is a more effective mutation detection method to reflect the complexity of river dynamic system evolution.

According to the statistics (Table 3), the annual sediment discharge decreased significantly after 1984 and 2008 at Wu Long station, especially after 2008. From 1984 to 2007, the sediment discharge decreased by 0.117.3 billion tons compared with 1960–1983, with a decrease of 50.73%. From 2008 to 2016, the sediment discharge decreased by 0.14 billion tons compared with 1984–2007, with a decrease of 83.33%.

**Table 3:** Annual average sediment discharge in various phases at Wu Long Station.

Year	Sediment discharge	Decrement compared with last phase (10^8^t/a)	Decline degree (%)
1960–1983	0.341		
1984–2007	0.168	0.173	50.73
2008–2016	0.028	0.14	83.33

## Discussions

### Sediment source

**Fig 5:**
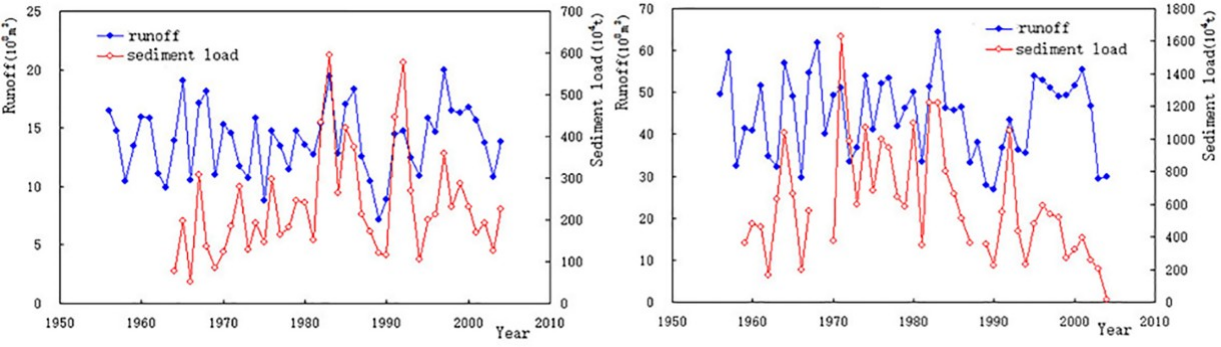
(a). Water and Sediment Variation at Yangchang Station Fig 5 (b). Water and Sediment Variation at Hongjiadu Station.

The strong sediment production area (sediment transport modulus greater than 1,000 t/ km^2^.a) mainly distributes in Sancha River and Liuchong River areas in the upper Wu Jiang River. Its area is less than 6.24% of the total area, but its sediment production reaches 19.68% of the total. Puding hydropower station located in the upper Sancha River was impounded in 1994. After the completion of the project, it intercepted almost all incoming sediment in the upper and middle reaches of Sancha River. The Hongjiadu hydropower station located in the upper Liuchong River was impounded in April 2004. After the completion of the project, it intercepted almost all incoming sediment from the Liuchong River. According to the analysis of the measured water and sediment data of Yangchang hydrological station in the upper Sancha River (Fig 5A) and Hongjiadu hydrological station in the upper Liuchong River (Fig 5B), it is found that the annual sediment discharge in the upper Sancha River changes little before and after the construction of Puding hydropower station, and the annual sediment discharge in the upper Liuchong River decreases significantly after the completion of the impoundment of Hongjiadu hydropower station.

According to the statistical analysis of water and sediment of various stations in Wu Jiang River Basin, the incoming water in the areas above Wujiangdu, the middle Wu Jiang River (Wujiangdu-Sinan interval) and the lower Wu Jiang River (Sinan-Wulong area) accounted for 30.1%, 25.1% and 44.8% of the total incoming water at Wu Long station, respectively. The sediment discharge mainly comes from the areas above Wujiangdu and Sinan-Wulong, accounting for 35% and 57.2% of the total sediment discharge at Wu Long station, respectively. The sediment discharge from Wujiangdu-Sinan is relatively small. Since the storage of the Wujiangdu hydropower station in 1980, the distribution of sediment transport areas in the Wu Jiang River Basin has changed significantly. The main manifestations are that the proportion of sediment transport in the areas above Wujiangdu has greatly decreased, the proportion of sediment transport in the middle Wu Jiang River has not changed much, and the proportion of sediment transport in the downstream areas has increased.

### Sediment transport characteristics

The annual distribution of sediment transport is very concentrated at Wu Long station. Sediment is nearly transported in flood season in the whole year. The sediment discharge covers 93.4% of the total during May to October in the whole year. That in major flood season from June to August and the maximum monthly sediment discharge cover 72.6% and 33.2% of that in the whole year respectively.

**Fig 6:**
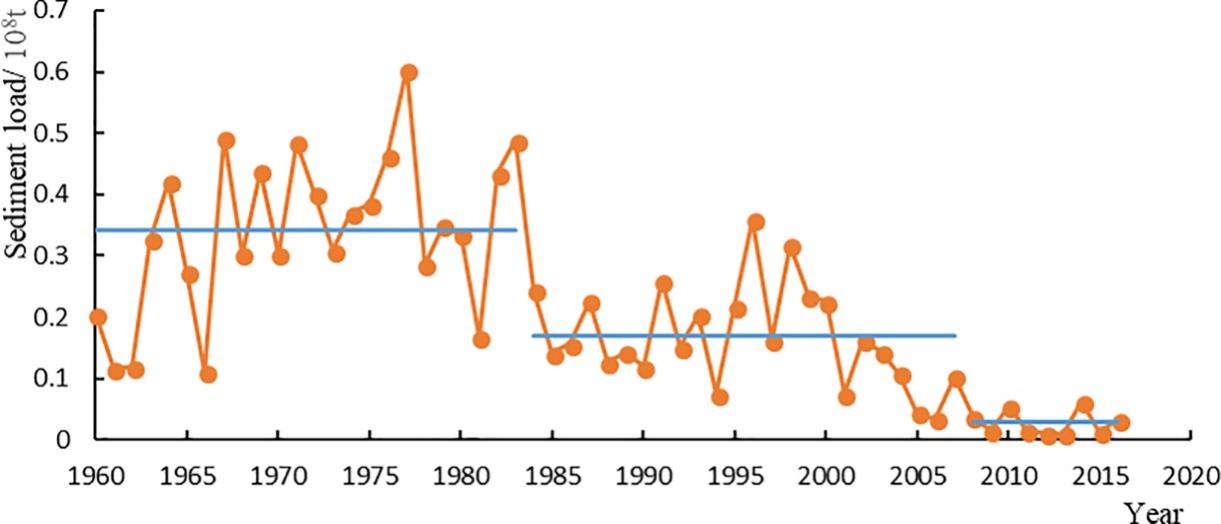
Annual sediment discharge at Wu Long Station (1960–2016).

**Fig 7:**
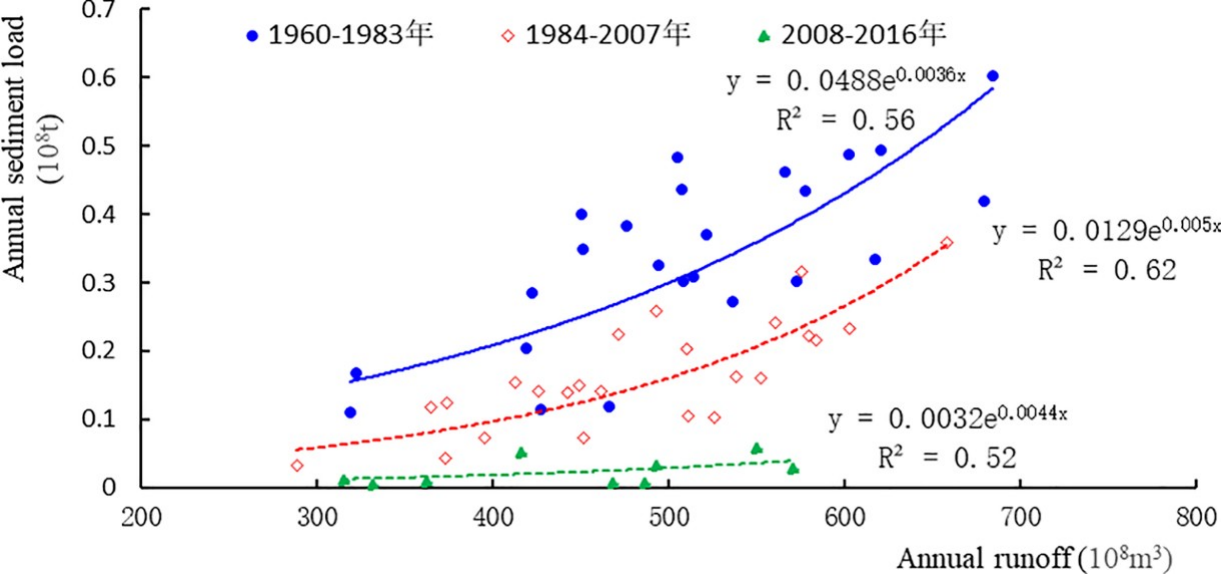
Runoff volume-Sediment discharge relation graph in various stages at Wu Long Station.

It shows that the annual sediment discharge is generally large from mid-1960s to 1980s during 1960 to 2016 at Wu Long station (Fig 6); while from mid-1980s to now, it greatly decreases. Especially after 1984 and 2008, the annual sediment discharge has an obvious mutation process. Conduct contrastive analysis on the relation schema of runoff—sediment discharge (Fig 7), and we can find that the inter-annual variation of sediment discharge is largely affected by the inter-annual change of runoff, which basically presents the characteristics of bigger water, larger sand amount, and smaller water, smaller sand amount. Generally, sediment discharge changes accordingly with the decrease and increase of runoff. Meanwhile, catastrophic flood leads to intense erosion of surface, and brings relatively large inter-annual change of sediment discharge. The catastrophic flood in 1977 increased the sediment discharge in the same year several times of the average value of long years.

## Influencing factors of sediment discharge

Factors affecting sediment production and sediment transport can be divided into natural and anthropogenic factors. Natural factors mainly include geology and geomorphology, soil and vegetation condition, and climate etc. Anthropogenic factors mainly include reservoir sediment retention, soil and water conservation, sediment increase in engineering construction and sand mining in river channels [27]. Among the above factors, the factors of geology, geomorphology, soil and vegetation (natural zonal vegetation) are relatively stable, and have little influence on the change of erosion and sediment production at different time. Climate change and anthropogenic factors usually have periodicity with different time scales, which are important factors affecting the change of sediment production and sediment transport in watershed erosion [28,29].

### Climate

Climate change mainly refers to the change of rainfall amount and distribution. Rainfall is the dynamic condition for sediment generation in earth surface. Its space-time (including time, impact area, intensity, duration etc.) distribution exerts a direct influence on sediment generation in the drainage basin. According to statistics during 1960 to 2016, the average annual rainfall is about 1,128 mm. From the statistics of annual average rainfall and runoff in the upstream of Wu Long Station in different time periods, we can see that they are in decrease tendency. And the annual rainfall during 1960 to 2016 was 1,154 mm, 1,120 mm during 1984 to 2007, and 1,078 mm during 2008 to 2016. The annual average runoff in different time periods were 51.04 billion m^3^, 48.27 billion m^3^, and 44.25 billion m^3^ (Table 4).

**Table 4:** Annual average rainfall and runoff at Wu Long Station.

Year	1960–1983	1984–2007	2008–2016
Areal rainfall/mm	1154	1120	1078
Runoff /10^8^m^3^	510.4	482.7	442.5

The decrease of rainfall in Wu Jiang drainage area is an important factor to cause decrease of annual sediment discharge. For example, in the continuous 5 years from 2009 to 2013 when there was lack of water, the runoff averagely reduced 103.2m^3^/s, which was 21.3%, and the sediment discharge droped drastically. According to the annual runoff—sediment discharge relation at Wu Long station (Fig 7), we can preliminarily calculate the sediment discharge reduction caused by decrease of runoff. Comparing with the time period during 1960 to 1983, the annual sediment discharge was 2 million t reduced due to reduction of runoff during 1984 to 2007, which covered 11.6% of the annual average total sediment reducing amount. Comparing with that during 1984 to 2007, it averagely reduced 1.2 million t during 2008 to 2016, which covered 8.6% of the annual average sediment reduction. To sum up, the loss of sediment discharge due to reduction of runoff covered about 10% of the reducing sediment at Wu Long station.

### Reservoir sediment retention

Wu Jiang River is the largest branch in the south shore of Yangtze River. Development of hydro-junctions in the basin is an important human activity affecting the change of water and sediment in the main and tributaries of the Wu Jiang River. According to incomplete statistics [30,31,32], during 1956 to 2005 there were 1,813 large, middle-sized and small reservoirs built in Wu Jiang River drainage basin (Table 5) with a total storage capacity of 12.098 billion m^3^. There are 7 large reservoirs with 10.052 billion m^3^ storage capacity, 42 middle sized reservoirs with 0.97 billion m^3^ capacity and 1,764 small reservoirs with 1.076 billion m^3^ capacity. During 2006 to 2016, there were 6 large reservoirs newly built in Wu Jiang drainage basin, with a total capacity of 9.911 billion m^3^, including Suofengying (in 2006), Pengshui (in 2009), Goupitan (in 2011), Silin (in 2011), Yinpan (in 2011) and Shatuo (in 2013).

**Table 5:** Statistics of reservoirs built in Wu Jiang River Basin.

Time Frame	Reservoir Group Summation		Large		Middle-sized		Small	
	Quantity	Total Storage (10^8^m^3^)	Quantity	Total Storage (10^8^m^3^)	Quantity	Total Storage (10^8^m^3^)	Quantity	Total Storage (10^8^m^3^)
1956~1990	1630	44.06	3	31.33	16	3.40	1611	9.33
1991~2005	183	76.92	4	69.19	26	6.30	153	1.43
2006~2016	334	102.86	6	99.11	9	2.26	319	1.49
1956~2016	2147	223.84	13	199.63	51	11.96	2083	12.25

During 1956 to 1990, except that Wujiangdu Power Station located in the main stream of the upstream of Wu Jjiang River, most of other middle sized and small reservoirs located in the tributary with relatively small sediment trapping amount. In the end of 1979 after the construction of Wujiangdu, river bed erosion adjustment exerted a great role in the downstream of the dam. Within the 4 years after water storage, it had an intense erosion adjustment, and the power station sediment trapping did not greatly affect the sediment amount at Wu Long station. But after 1984, due to the gradually weakening erosion intensity in the downstream of Wujiangdu power station, the influence of the sediment trapping increased gradually at Wu Long station.

During 1956 to 1983, the total sediment trapping amount of the reservoir group was 0.107 billion m^3^ (among which the total amount reached 75.6 million t during 1972 to 1983 in Wujiangdu power station), and the annual average sediment trapping amount was about 4.4 million t, which exerted a little influence on the sediment turnoff at Wu Long station. During the period of “7th Five Years Programs for Science and Technology Development of China”, Shi Guoyu adopted multi-dimensional dynamic grey system theory and created the sediment production and sediment transport formula [33] in Wujiang drainage area (at Wu Long station):

$$X_{1}(k)=0.801X_{1}(k-1)+1.59X_{2}(k)-0.034X_{3}(k)$$

The sediment trapping coefficient of the reservoir group in this drainage basin is calculated to be 0.034 after analysis. Thus, we can see that the reducing sediment caused by reservoir group sediment trapping covers 3.4% of the sediment trapping amount of reservoir group at Wu Long station. That is, the sediment reduction caused by reservoir group is about 200,000 t at Wu Long station, which covers 0.6% of the average sediment discharge in the same period. In the early stage after reservoir completion, the river bed erosion adjustment amount is relatively little in the downstream of the dam. The erosion quantity in the downstream can basically offset the reservoir sediment trapping, thus it exerts little influence on the sediment discharge at Wu Long station.

During 1984 to 1990, the alluvial sediment reached 0.127 billion m^3^ (0.147 billion t) in Wujiangdu power station, with the annual average sediment trapping amount to be 21 million t. The average annual sediment discharge reached 16.6 million t during 1984 to 1990 at Wu Long station, which was 16 million t and 19.1 million t less than that during 1955 to 1979 and during 1980 to 1983 respectively, covering 49.1% and 53.5% (corresponding runoff reduced 5.9 billion m^3^ and 9.5 billion m^3^, covering 11.9% and 17.9%. If to consider the influence of inconsistent runoff, and to calculate with the average sediment concentration during 1955 to 1979 and during 1980 to 1983 as 0.660kg/m^3^ and 0.674 kg/m^3^, the sediment discharge can actually reduce 12.1 million t, and 12.7 million t (average 12.4 million t). Then the influence coefficient of the sediment trapping in Wujiangdu power station on sediment is 0.59 at Wu Long station.

Therefore, as an approximate estimation, the sediment discharge reduced by sediment trapping of reservoir group is 2.6 million t during 1956 to 1990 in Wujiangdu power station, the comprehensive coefficient is 0.29.

Since 1990s, there was a great change in sediment production and transport in Wu Jiang drainage basin. The mid and downstream of main stream of Wu Jiang River is mountainous watercourse. After clear-water washing in the preliminary stage of water storage in Wujiangdu power station, the fine-grained sediment in the downstream of watercourse is basically all washed out. Thus, the river-bed erosion adjustment amount caused by reservoir sediment trapping in the downstream greatly decreases with the time.

Reservoir sediment trapping during 1991 to 2005 mainly depended on large power stations built in the main stream of Wu Jiang River. Due to most of mid and small reservoirs located in the end of tributary or drainage, which were far from Wu Long station, the influence of sediment trapping on sediment discharge can be neglected at Wu Long station. From the sediment discharge change along the watercourse before and after water storage in Wujiangdu power station, we can see that the incoming sediment amount from the downstream of Wujiangdu power station to the Jiangjiehe was not great. The drainage area in this section was 14,468 km^2^, covering 34% of the control basin area of Jiangjiehe hydrometric station. But its incoming sediment amount was just 2 million t before water storage in Wujiangdu power station, covering 16% of the sediment discharge of Jiangjiehe station, which shows that above 80% of sediment discharge in Jiangjiehe station before water storage came from the upstream of Wujiangdu. The sediment discharge were respectively 189,000 t and 3.03 million t in Wujiangdu and Jiangjiehe station during 1991 to 2004 after water storage in Wujiangdu power station. It shows that after water storage, the sediment discharge in Jiangjiehe station mainly came from the section in the downstream of Wujiangdu power station. Sediment discharge in Wujiangdu just covered 5%.

After water storage in Wujiangdu, its sediment trapping leads to great decrease of downstream sediment discharge in the downstream. For example, the average suspended sediment transport capacity was about 15.8 million t during 1956 to 1978 in Wujiangdu hydrometric station before water storage in power station. The average sediment discharge was just 189,000 t during 1991 to 2004 after water storage (including sediment trapping of Dongfeng power station in the upstream). Water storage and sediment trapping leads to a great decrease of sediment discharge in the downstream at Wujiangdu power station. According to the design materials of each hydropower station in main stream of Wu Jiang River [34], it was reduced to 3.68 million t after water storage of Wujiangdu, which was 81% reduced. But the annual average sediment discharge were 15.7 million t and 610,000 t respectively before and after water storage in Wujiangdu station. It shows that before water storage in Wujiangdu power station, the incoming sediment discharge in the area between Wujiangdu and Silin power station was 3.3 million t, and that was 3.01 million t after water storage. Then the average annual erosion adjustment amount after water storage in power station was just 290,000 t. It’s known after the above analysis that during 1980 to 2005, the total sediment trapping amount was 390.0 million m^3^ in power stations like Wujiangdu, Dongfeng and Puding, with an average annual sediment trapping amount to be 15 million m^3^ (17.4 million t). Therefore, the function coefficient of Wujiangdu power station at Silin power station is 0.983.

Shatuo hydropower station dam is 250.5 km away in the downstream. Before water storage in Wujiangdu, its average sediment discharge was 20.50 million t for many years. After water storage in Wujiangdu, it’s reduced to 6.01 million t, which was 71% reduction (annual average sediment discharge were respectively 15.7 million t and 610,000 t before and after water storage in Wujiangdu).

Before water storage in Wujiangdu power station, the incoming sediment discharge was 4.8 million t between Wujiangdu and Shatuo power station. After water storage, the incoming sediment amount was 5.4 million t. Then the annual average erosion adjustment amount was just 600,000 t after water storage in the power station. Therefore, the function coefficient of Wujiangdu at Shatuo power station is 0.966.

Wu Long station locates in about 440km away in the downstream of Shatuo power station. It can be calculated that the function coefficient of sediment trapping of large hydropower stations was about 0.9 in the upper and middle stream of Wu Jiang River at Wu Long station. In conclusion, the sediment reducing amount in drainage basin caused by comprehensive sediment trapping of reservoirs built after 1990 covered 90% of sediment reducing amount in the upper and middle stream of Wu Jiang River at Wu Long station. The reservoir group sediment trapping is the main factor to lead to decrease of sediment discharge in Wu Jiang River drainage basin, and is the most direct cause for mutation of sediment discharge at Wu Long station.

### Soil and water conservation

Bijie in the upstream of Wu Jiang River suffers from relatively serious water and soil loss in Wu Jiang drainage basin. Its comprehensive treatment of water and soil conservation also mainly focuses on this area.

Since 1989, Bijie is listed into “comprehensive treatment project for key prevention areas of soil and water conservation in the upper Yangtze River”. The 4 counties, Bijie, Dafang, Weining and Hezhang, were listed in the phase I treatment project (1989 to 1993), covering a treatment area of 3.3063 million mu (2,204 km^2^). From 1989 to 1994, on the basis of comprehensive control of four counties in the first phase of the project, Qianxi County and Jinsha County were added to carry out comprehensive control of soil and water loss in small watershed in Bijie District. The control area was 253.17 km^2^, and the amount of soil erosion in the watershed was reduced from 3.12 million t/year in 1990 to 1.02 million t/year in 1994, with the erosion reduction rate reaching 67%. From 1994 to 1998, Bijie area carried out the comprehensive management of small watershed in the third phase of "Changzhi" project. After the treatment, the area of soil erosion decreased from 1,909 km^2^ in 1994 to 1,016 km^2^ in 1998, while the area of soil erosion above intensity decreased from 647.53 km^2^ to 206.37 km^2^, a decrease of 62%.

Soil erosion amount decreased from 9.05 million t/ year in 1994 to 2.97 million t/ year in 1998, with an annual average erosion reduction as 6.08 million t/year, 67% corrosion rate deduction. And the average annual sediment trapping was about 5.25 million t in soil and water conservation projects like soil reservation cultivation etc. During 2001 to 2004, the comprehensive treatment area of soil and water loss was 278.4 km^2^ in large demonstration area project of Bijie road soil and water conservation ecological environment construction.

Therefore, after soil and water conservation measures were taken in Bijie during1989 to 2004, the annual average erosion reduction amount was about 10 million t. According to statistics analysis during 1957 to 1990, the sediment delivery ratio in Bijie was about 0.2 to 0.3. So the sediment reducing amount after soil and water reservation in Bijie is 2 to 3 million t(average 2.5 million t).

The annual average runoff were 4.45 billion m^3^ and 4.55 billion m^3^ respectively before and after the year 1990 in Hongjaidu Station. The annual average sediment discharge were 6.85 million t and 4.49 million t (from 1991 to 2003), and sediment discharge reduced 2.36 million t. In 2004, due to the influence of sediment trapping of Hongjiadu power station, its sediment discharge was just 110,000 t. It shows from the comparison of before and after taking water conservation measures, the relation between runoff and sediment discharge, and between rainfall and sediment discharge had apparent change. Its water conservation measures reduced about 2.6 to 3.2 million t averagely (an average of 2.9 million t). Thus, the sediment reducing amount was about 2.9 million t in Liuchonghe drainage basin after the soil and water conservation measures.

To sum up, the annual average sediment reducing amount is about 2.5 to 2.9 million t (an average of 2.7 million t) in Bijie in the upper Wu Jiang River (Liuchonghe drainage basin). It mainly reflects in that decreased incoming sediment exerts no big an influence on the reduced sediment discharge in Dongfeng and Puding Power station.

It deserves to point out that the implementation of “Changzhi” project improves the soil and water loss in Wu Jiang drainage area. It exerts a certain role of water storage and sediment trapping under normal rainfall conditions. But the distribution of annual sediment transport is more and more concentrated in flood season in Wu Jiang River, and 88% of annual precipitation is concentrated in April to October. The precipitation accounts for about 70% in May to September of the whole year. The monthly precipitation accounts for the percentage of the whole year, with the largest proportion in June. At the same time, the maximum monthly sediment discharge has also increased considerably, and the benefits of water and sediment conservation measures under rainstorm conditions deserve further study.

## Discussion of results

This article introduces a new method for mutation detection, the approximate entropy method (ApEn), which based on the complexity of time series. Using this method to detect the mutation of average annual sediment discharge from 1960 to 2016 at Wu Long Station, and compared with the results of double cumulative curve method, B-G segmentation algorithm and sliding t test method. It can be seen that although B-G segmentation algorithm method and sliding t test method can also detect the corresponding structural mutations, it can’t reflect the dynamic structural differences and complexity before and after the mutations, and the detection results depend on the selection of time scale, so the detected mutation points have multi-time scale characteristics. While, the ApEn method can reflect the degree of self-similarity of time series in patterns and the possibility of generating new patterns when the dimension m changes. Besides, the ApEn method just wants to distinguish the complexity of time process from the statistical point of view, to represent the difference or change of dynamic system, so it has the advantage of shorter time series length needed for calculation. Therefore, the ApEn method has obvious advantages in mutation detection of annual sediment discharge in river basins, and it is a more effective mutation detection method to reflect the complexity of river dynamic system evolution.

## Conclusions

Approximate entropy (ApEn) method is adopted for annual mutation detection on the annual sediment discharge at Wu Long Station. And the main conclusions are drawn as below after analysis:

1. The approximate entropy method is an effective method to detect themutation of annual sediment discharge in rivers. By processing the low frequency sequence, the mutation information of relatively large scale sediment discharge in rivers can be distinguished.
2. Compared with 1960–1983, the sediment transport decreased by 17.3 million t and 31.3 million t respectively from 1984 to 2007 and from 2008 to 2016 in Wu Jiang River. Reservoir sediment retention is the most important factor to decrease of sediment discharge in Wu Jiang drainage basin. The sediment reducing amount caused by reservoir trapping covers 90% of sediment reduction amount at Wu Long station. The decrease in runoff caused by climate change (rainfall) covers 10% of sediment trapping amount. And the soil and water conservation measures in Bijie exert a small influence on the decrease of sediment discharge in the upper Wujiang River.

With the continuous development of hydropower development and soil and water conservation projects in the basin, human activities have become an important factor affecting the relationship between runoff and sediment transport. The interaction and coupling mechanism of rainfall-sediment production -sediment transport-reservoir sediment retention-sediment reduction measures need to be further studied.

## Supporting information

S1 File (DOCX)
