# Supplementary material for: Mutation analysis of annual sediment discharge at Wu Long station in Wu Jiang River Basin from 1960 to 2016
Source: PLoS One. 2019 Dec 16;14(12):e0225935. doi: 10.1371/journal.pone.0225935 (PMC6913992; doi:10.1371/journal.pone.0225935)
Supplement: S1 File — (DOCX) [file pone.0225935.s001.docx]

**S1 file:**

**Fig 1. Distribution chart of main hydrometric stations and large hydropower stations in Wu Jiang Basin**

**(TIF)**

**S2 file:**

**Fig 2. ApEn value process line of annual sediment discharge at Wu Long Station**

**(TIF)**

**S3 file:**

**Fig 3. Dobule cumulative graph of annual runoff and sediment discharge at Wu Long Station**

**(TIF)**

**S4 file:**

**Fig 4. Sliding T-test of annual sediment discharge at Wu Long Station**

**(TIF)**

**S5 file:**

**Fig 5A. Water and Sediment Variation at Yangchang Station**

**(TIF)**

**Fig 5B. Water and Sediment Variation at Hongjiadu Station**

**(TIF)**

**S6 file:**

**Fig 6. Annual sediment discharge at Wu Long Station (1960-2016)**

**(TIF)**

**S7 file:**

**Fig 7. Runoff volume-Sediment dsicharge relation graph in various stages at Wu Long Station**

**(TIF)**

**Protocols：**

Manuscript Citation: dx.doi.org/10.17504/protocols.io.544g8yw

Fig 1: dx.doi.org/10.17504/protocols.io.yckfsuw

Fig 2: dx.doi.org/10.17504/protocols.io.ycmfsu6

Fig 3: dx.doi.org/10.17504/protocols.io.ycnfsve

Fig 4: https://protocols.io/view/fig-4-545g8y6

Fig 5: dx.doi.org/10.17504/protocols.io.ycpfsvn

Fig 6: dx.doi.org/10.17504/protocols.io.ycrfsv6

Fig 7: dx.doi.org/10.17504/protocols.io.ycsfswe

Table 1: dx.doi.org/10.17504/protocols.io.yctfswn

Table 2: dx.doi.org/10.17504/protocols.io.ycufsww

Table 3: dx.doi.org/10.17504/protocols.io.ycvfsw6

Table 4: dx.doi.org/10.17504/protocols.io.ycwfsxe

Table 5: dx.doi.org/10.17504/protocols.io.ycxfsxn
